# Attitudes towards Donor Breast Milk in an Inner City Population

**DOI:** 10.1155/2019/3847283

**Published:** 2019-01-01

**Authors:** Abhinav Pal, Kultida Soontarapornchai, Lawrence Noble, Ivan Hand

**Affiliations:** Division of Neonatology, Department of Pediatrics, Kings County Hospital Center, SUNY Downstate School of Medicine, Brooklyn, New York 11203, USA

## Abstract

*Objective. *The purpose of our study was to identify attitudes towards donor breast milk in our population and identify barriers to its acceptance.* Methods. *The study sample was comprised of a convenience sample of 174 postpartum women. A questionnaire consisting of demographic information and 12 questions relating to attitudes and understanding of donor breast milk was administered.* Results. *Among the mothers surveyed, 34% were aware of the use of donor breast milk and donor milk banks. 62% of mothers preferred the use of formula compared to donor breast milk if they were unable to provide their own breast milk. Educational level did play a role with 64% of mothers with education beyond high school believing that donor breast milk was beneficial for newborns as opposed to 46% with a high school education or less (p=0.02). US born mothers were more likely to have heard about donor breast milk (47% versus 29%, p=0.025) than foreign born mothers although they were less likely to believe it was a better option for feeding than formula (22.7% versus 43%, p=.016). Mothers with infants in the NICU were more likely than mothers of well babies to accept milk from a milk bank rather than a relative or friend (81% versus 39%, p≤0.001).* Conclusion*. Although the beneficial effects of donor breast milk are well established in the scientific community, there remains a lack of awareness and a major discrepancy in the understanding and acceptance of it within our community. Education on the benefits of mother's own milk as well as donor breast milk and milk banks is an important public health initiative needed to increase acceptance of human milk as the optimal form of nutrition in infants.

## 1. Introduction

The American Academy of Pediatrics has reaffirmed its recommendation that infants be exclusively breastfed for the first 6 months of life and that human milk is the optimal form of nutrition for infants [1]. Breastfeeding has been associated with significant improvements in outcomes including decreased rates of infection, obesity and improved neurodevelopment [2–6]. Preterm infants fall into an especially critical category with respect to their susceptibility to infection, necrotizing enterocolitis, feeding intolerance and generally increased morbidity and mortality [7–10]. When mother's own milk is unavailable the use of donor human milk is recommended [1, 11]. Donor human milk has been shown to offer several advantages over formula feeds, including lower rates of infection and feeding intolerance [12, 13].

Despite the beneficial effects of human milk, significant racial and ethnic differences remain between groups [14, 15]. Black mothers' intentions to breastfeed as well as initiation and duration of breastfeeding were significantly less than white mothers. Poverty also was found to be a significant mediator of differences in breastfeeding initiation and intent [15]. As we sought to introduce donor breast milk into our inner city NICU we chose to study the attitudes of mothers towards its use in our population. The purpose of our study was to identify the attitudes towards donor breast milk in our community and identify barriers to its acceptance.

## 2. Methods

This study was conducted between July and August 2015 at Kings County Hospital, located in Brooklyn, NY. The study sample was comprised of a convenience sample of 174 postpartum women. A questionnaire consisting of demographic information and 12 questions relating to attitudes and understanding of donor breast milk was administered by one of the authors (Table 1). The survey was taken on the postpartum floor within 48 hours of delivery. Data was analyzed using SPSS 20. Descriptive statistics were used to summarize the data and identify characteristics of the mothers and newborns. Chi-square and Student t-test were used for analysis. The study received approval from the Institutional Review Board at SUNY Downstate College of Medicine.

## 3. Results

### 3.1. Demographics

There were 174 mothers who were interviewed for the study. The age range of the mothers was 18- 45 years with a mean of 29 years. Racial/ethnic identification was based on mothers' self-report as either non-Hispanic white, non-Hispanic black, or Hispanic. Over 95% of our population identified themselves as non-Hispanic black, with the majority of mothers having been born in the Caribbean. Of the group of mothers surveyed, only 25% of them were born in the USA, although 70% have been living in the United States for more than a year. (Table 2)

Among the mothers surveyed, 34% were aware of the use of donor breast milk and donor milk banks. 62% of mothers preferred the use of formula compared to donor breast milk if they were unable to provide their own breast milk. Among those who preferred formula, reasons for preference of formula were varied with 88% not wanting to give someone else's milk to their baby, 77% fearing transmission of infection, 30% citing increased cost and lack of insurance reimbursement, and 4% felt it violated their religious beliefs. Mothers who preferred formula, compared to others, felt that donor breast milk had less nutrients than formula (9% versus 1.5%, p=0.04), was more likely to transmit infections to infants (69% versus 50%, p=0.01), had no immunological benefit (27% versus 14%, p=0.04), and did not offer more advantages than disadvantages (64% versus 18%, p ≤0.001). Educational level did play a role with 64% of mothers with education beyond high school believing that donor breast milk was beneficial for newborns as opposed to 46% with a high school education or less (p=0.02).

In our population, 41% of the mothers questioned had previously breastfed their infants. Of those who had previously breastfed their infants the mean length of feeding was 11 months. Mothers more than 30 years old were more likely to breastfeed for more than 3 months (58% versus 30%, p≤0.01) as were mothers who were married (51% versus 33%, p= 0.02). Mothers who had breastfed for more than 3 months were more likely to have the opinion that donor breast milk had more advantages than disadvantages (63% versus 47%, p=.029).

### 3.2. Foreign Born

Mothers born in the United States were less likely to have previously breastfeed for more than 3 months (9% versus 51%, p≤0.001) than foreign born mothers. US born mothers were more likely to have heard about donor breast milk (47% versus 29%, p=0.025) than foreign born mothers although they were less likely to believe it was a better option for feeding than formula (22.7% versus 43%, p=.016). Foreign born mothers were more likely to feel that breast milk from donor banks was safe (38% versus 11.1%, p=0.003), did not transmit infection (43.7% versus 22.7%, p=0.013) and its benefits outweighed its risks (58.4 versus 38.6%, p=0.023) than mothers born in the United States.

### 3.3. Infant Status

There were 130 infants in the well-baby nursery and 44 admitted to the NICU. Mothers with infants in the NICU were more likely than mothers of well babies to accept milk from a milk bank rather than a relative or friend (81% versus 39%, p<0.001). However, the majority of mothers with sick infants in the NICU, 56%, believed that donor milk banks are not a safe source of milk. In contrast, 72% of mothers of well babies believed that donor milk banks are not a safe source of milk (p=0.043). There was no significant difference for other issues related to the benefits of breast milk over formula.

## 4. Discussion

The goal of this study was to identify the attitudes towards donor breast milk in our population prior to initiating a donor breast milk program in our hospital. While a number of major studies have proved the efficacy of donor breast milk, the attitude of mothers towards it varies across the population worldwide. We found that only 34%, one-third of our postpartum mothers, were aware of donor breast milk. A recent study in Turkey revealed that 90.6% of women who gave birth were unaware of breast milk banking. After educational efforts, 64% of them were open to donation, although 36.3% saw it as an issue from a religious standpoint and almost 30% of them saw it causing social and moral problems [16]. A recent study in Ohio showed a high awareness of milk sharing (77%) among mothers. It also showed that education level of mothers was directly proportional to their chances of donating milk and that mothers who delivered preterm were more likely to consider using donor human milk for their babies [17]. Our study demonstrated a similar tendency among mothers with a higher educational level to believe donor breast milk was beneficial as well as among women whose infants were admitted to the NICU.

Our study population was quite homogenous, with over 95% identifying as non-Hispanic black. Although there have been many studies related to ethnicity and breastfeeding [18, 19] there have been few focusing on donor milk in this population. Delfosse [20] described an intervention replacing formula with donor human milk in 2 urban NICUs. Although successful, the study only surveyed 13 women prior to initiation and their non-Hispanic black population ranged from 24 to 43%.

As has been reported, foreign born mothers have higher rates of breastfeeding than the US born population, most likely due to less maternal acculturation [14]. Although foreign born mothers were less aware of donor milk banks, they demonstrated more trust in donor milk and felt it was more beneficial than their US born counterparts.

The strength of this study is that it one of a very few focusing on attitudes towards donor milk in this population and highlights the general lack of education. Our study may not be generalizable to other populations that are less racially homogenous and of different socioeconomic levels.

There appears to be a significant amount of misinformation in our population concerning the safety of donor breast milk and its advantages over formula. This may reflect a general lack of familiarity with the benefits of breast feeding in our population. Thus, while the beneficial effects of donor breast milk seem to be well established in the scientific community, there remains a lack of awareness and a major discrepancy in the understanding and acceptance of it within our community. Introduction of donor milk in the NICU, similar to any medical treatment, requires a thorough explanation to the parents of the risks and benefits of human milk for the preterm infant in order to obtain their trust and consent. Education on the benefits of mother's own milk as well as donor breast milk and milk banks is an important public health initiative needed to increase acceptance of human milk as the optimal form of nutrition in infants.

## Figures and Tables

**Table 1:** Donor Breast Milk Questionnaire.

(1) Have you heard about the availability of donor breast milk? Y/N
(2) If you do not have enough breast milk, donor breast milk is a better option for your baby than formula. Select the best option. Y/N
(a) Why do you prefer formula instead of donor milk? (free text)
(3) If you had to choose donor breast milk, which donor would you select? Relative/friend or Milk Bank
(4) If Donor Breast Milk was available for your baby, do you think you would stop pumping your own milk: Y/N
(5) Donor breast milk contains more nutrients than formula milk. Y/N
(6) Breast milk donation bank in the USA is safe and trustworthy. Y/N
(7) Donor breast milk may transmit infection(s)/disease(s) from the donor to your baby. Y/N
(8) Donor breast milk provides some immunological benefits (immunity/resistance to infections) that can reduce the rate of infections Y/N
(9) Donor breast milk may increase the risk of infants having allergy. Y/N
(10) Overall, Donor breast milk has more benefits than disadvantages? Y/N
(11) Would your attitude toward Donor breast milk be changed, if you learn more about Donor breast milk? Y/N

**Table 2:** Sociodemographic data of mothers (n=174).

Variables	Frequency	Percentage
*Age of mothers ( in years)*		
<20	5	2.8
20-30	97	55.7
31-40	67	38.5
41-45	5	2.8
*Mothers' marital status*		
Single	99	57
Married/Living together	75	43
*Mothers' religion*		
Christian	152	87
Muslim	12	7
Other	10	6
*Mother educational status*		
High School	96	55
Some college or associate's degree	78	45
*Employment*		
Unemployed	129	74
Employed	45	26
*Nativity Status*		
US born	43	25
Foreign born	131	75
*Infant Status*		
Well baby	130	75
NICU	44	25

## Data Availability

The data used to support the findings of this study are available from the corresponding author upon request.
